# Emergency criteria and lifesaving intervention windows for high-risk critical congenital heart disease: a perspectives

**DOI:** 10.3389/fcvm.2026.1784096

**Published:** 2026-06-15

**Authors:** Brian Mendel, Edoardo Zancanaro, Inga Voges, Raymond N. Haddad

**Affiliations:** 1 Department of Cardiology and Vascular Medicine, National Cardiovascular Center Harapan Kita, Universitas Indonesia, Jakarta, Indonesia; 2 Heart and Vascular Institute, Brigham and Women's Hospital, Mass General Brigham, Harvard Medical School, Boston, MA, United States; 3 Department of Cardiac Surgery, San Raffaele Hospital, Milan, Italy; 4 German Centre for Cardiovascular Research (DZHK), Kiel, Germany; 5 Department of Congenital Heart Disease and Pediatric Cardiology, University Hospital Schleswig-Holstein, Kiel, Germany; 6 Filière des Cardiopathies Congénitales Enfant Adultes, Hôpital Marie Lannelongue, Centre Constitutif du Réseau Maladies Rares Malformations Cardiaques Congénitales Complexes-M3C, Hôpitaux Saint Joseph et Marie Lannelongue, Le Plessis Robinson, France

**Keywords:** critical congenital heart disease, immediate, intervention window, semi-urgent, urgent

## Abstract

Critical congenital heart disease (CCHD) is a major cause of neonatal cardiovascular instability and early mortality. Delays in recognition and definitive intervention frequently result in preventable end-organ injury or death. This perspectives proposes a physiology-based framework defining emergency clinical criteria and time-sensitive intervention windows for critical CHD to support rapid and effective decision-making.

## Introduction

Critical congenital heart disease (CCHD) comprises a heterogeneous group of cardiac malformations characterized by the inability of the cardiovascular system to adequately transition from fetal to postnatal circulation within the first days to weeks of life (1). These lesions frequently manifest during the neonatal period with rapid clinical deterioration, often triggered by ductal closure or failure of physiologic adaptation. Without timely recognition and intervention, affected infants may develop severe hypoxemia, cardiogenic shock, heart failure, or death (2, 3).

To improve early detection, universal newborn screening for CCHD using pulse oximetry was recommended internationally in 2011 and has since proven to be both effective and cost-efficient. Population-based analyses have demonstrated a significant reduction in infant mortality following its implementation, including a reported 33% decline in deaths attributed to CCHD (4–6). Nevertheless, despite advances in prenatal diagnosis, neonatal intensive care, and surgical techniques, delayed recognition and intervention remain major contributors to morbidity and mortality. A substantial proportion of infants with critical lesions are still diagnosed after hospital discharge, particularly those with left ventricular outflow tract obstruction such as critical coarctation or aortic arch interruption (3).

**Figure 1:**
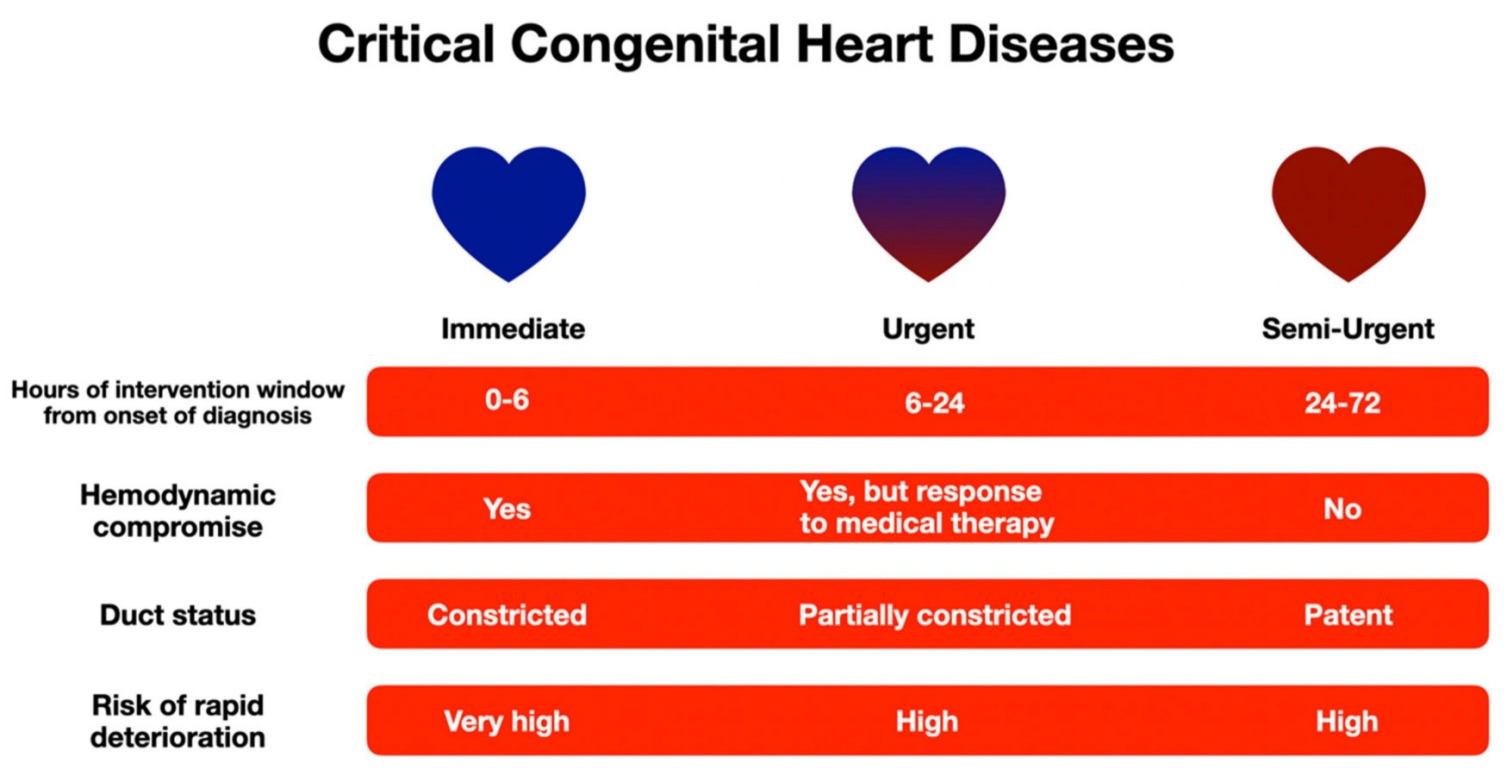
Interventional window for critical congenital heart diseases proposed by Mendel et al.

Congenital heart disease is generally considered a chronic condition requiring lifelong management. However, a subset of patients with CCHD experience acute and rapidly progressive physiologic deterioration that demands urgent stabilization rather than elective lesion-specific repair (1–6). Current clinical guidelines primarily focus on anatomical diagnosis and definitive surgical management (7). In emergency settings, especially in centers without immediate access to tertiary congenital heart services, clinical deterioration is more often driven by physiologic collapse than by precise anatomical categorization. In such scenarios, life-saving palliative interventions aimed at restoring systemic or pulmonary blood flow, improving intercirculatory mixing, or correcting life-threatening conduction disturbances become critical bridges to definitive therapy (see Figures 1).

Given the substantial advances in interventional and surgical therapies over the past two decades, there is an increasing need to reconsider how emergency care for CCHD is conceptualized and delivered. Rather than focusing solely on anatomical diagnosis, a physiology-based framework that emphasizes rapid stabilization and timely catheter-based rescue interventions may be essential to improve outcomes. In this perspective, we discuss the concept and practicality of emergency, life-saving palliative interventions in critical congenital heart disease and explore their potential role as a “door-to-balloon” paradigm in the management of acutely deteriorating patients.

While a physiology-based framework provides a practical approach for rapid decision-making in unstable patients, it has important limitations. Certain critical lesions may not respond to standard stabilization strategies. For example, total anomalous pulmonary venous return (TAPVR), particularly when obstructed, may present with profound hypoxemia and circulatory collapse and does not improve with prostaglandin therapy. In such cases, survival depends on rapid recognition, prompt transfer to a tertiary center, and access to advanced support such as extracorporeal membrane oxygenation or emergent surgical repair.

Furthermore, recognition of physiologic deterioration alone is insufficient without a targeted understanding of the underlying anatomy to guide intervention. In emergency settings, a focused or goal-directed echocardiographic assessment may be more practical than a complete diagnostic study. Rapid identification of key features, such as ductal-dependent systemic circulation requiring prostaglandin infusion (e.g., interrupted aortic arch) or transposition physiology with restrictive atrial communication requiring urgent balloon atrial septostomy, can facilitate timely and appropriate intervention while avoiding delays associated with comprehensive imaging.

## Definition of emergency CCHD

Emergency CCHD is defined as congenital heart disease associated with immediate or impending cardiovascular instability requiring urgent medical stabilization and palliative catheter-based or surgical intervention within a limited and clearly defined time window. This definition emphasizes physiologic vulnerability rather than anatomic complexity.

## The importance of critical CHD identification

Emergency presentation in neonates with critical congenital heart disease (CHD) is typically characterized by rapid clinical deterioration resulting from systemic hypoperfusion, respiratory failure, or progressive metabolic derangement (8). Hemodynamic instability may manifest as cardiogenic shock, systemic hypotension, or low cardiac output syndrome, often accompanied by significant metabolic acidosis. Respiratory compromise frequently presents as profound cyanosis that is unresponsive to supplemental oxygen, episodes of apnea, or respiratory failure requiring mechanical ventilation. These manifestations are usually driven by underlying cardiac pathophysiology, including critical valvar or vascular obstruction, restrictive intracardiac mixing, hemodynamic decompensation following ductal closure, or clinically significant arrhythmias. As circulatory compromise progresses, end-organ dysfunction may develop, involving the renal, neurologic, hepatic, or gastrointestinal systems, which reflects advanced systemic hypoperfusion and necessitates immediate intervention (4, 5, 7, 8).

Early identification of critical CHD through prenatal diagnosis plays a crucial role in improving clinical stability after birth. Prenatal detection enables prompt initiation of prostaglandin therapy to maintain ductal patency and allows for coordinated delivery planning at specialized centers, thereby reducing the risk of hemodynamic deterioration before neonatal cardiac intervention (9, 10). In contrast, postnatal diagnosis of critical CHD is associated with a higher likelihood of preoperative instability and has been linked to an increased risk of brain injury and less optimal brain maturation during the neonatal period compared with cases detected prenatally (11).

An additional practical consideration is the availability of prostaglandin E1 (PGE1), which remains a cornerstone of initial stabilization in duct-dependent lesions. Due to its relatively high cost, short shelf-life, and storage requirements, access to PGE1 may be limited in some healthcare settings, particularly in low- and middle-income regions. This constraint underscores the importance of early recognition, timely referral, and the development of context-specific management strategies when standard pharmacologic stabilization is not immediately available.

Neurologic complications are an important concern in this population. Seizures may occur as a consequence of underlying brain injury, may themselves contribute to further neuronal damage, or may result from a combination of both mechanisms. In a multicenter study, seizures were observed in approximately 7.4% of neonates following cardiac surgery; notably, the majority of these events were identified only through continuous electroencephalographic monitoring, highlighting the importance of vigilant neurologic surveillance (12, 13).

Cardiac arrest represents another severe complication in children with cardiovascular disease. The incidence of cardiac arrest requiring resuscitation is approximately 7 per 1,000 hospitalizations in this population, which is more than ten times higher than the rate observed in children hospitalized without cardiovascular disease. Cerebral hypoperfusion during cardiac arrest can lead to hypoxic–ischemic brain injury, a complex process involving oxidative stress, reperfusion injury, and ongoing neuronal cell death through necrosis and apoptosis. Importantly, these pathophysiological processes may continue for weeks after the initial arrest, contributing to long-term neurologic morbidity (14–16).

## Intervention window classification

Catheter-based interventions (e.g., balloon atrial septostomy or inter-atrial stenting, ductal stenting, and right ventricular outflow tract stenting) play an important role in the management of critical CHD by addressing key physiologic derangements (17–19). Balloon atrial septostomy is often performed emergently to improve intercirculatory mixing in patients with restrictive atrial communication (17). In contrast, ductal stenting and right ventricular outflow tract stenting are typically performed after initial stabilization, including prostaglandin E1 infusion when indicated, and serve as either bridging or alternative strategies to surgical palliation (18, 19). Balloon valvotomy and other catheter-based interventions may relieve critical obstruction and facilitate stabilization prior to definitive repair (19, 20). The choice and timing of intervention are guided primarily by physiologic status and clinical urgency rather than anatomy alone.

The *Immediate* intervention window, defined as the first six hours following recognition, includes patients at imminent risk of cardiovascular collapse or cardiac arrest. These patients typically present with profound hypoxemia or shock related to restrictive atrial level mixing, closing ductus arteriosus, or critical outflow obstruction. Survival depends on immediate initiation of prostaglandin E1, aggressive cardiorespiratory support, and emergent catheter-based or surgical intervention.

The *Urgent* intervention window spans six to twenty-four hours and applies to patients who remain hemodynamically unstable but partially responsive to medical therapy. Although temporarily stabilized, these patients carry a high risk of rapid deterioration. Early definitive intervention during this period is required to prevent progression to irreversible end-organ injury.

The *Semi-Urgent* intervention window extends from twenty-four to seventy-two hours and includes patients who are clinically stable at presentation but harbor lesions with a high likelihood of deterioration. These patients require close monitoring and early planned intervention during the same hospitalization, as delayed treatment may convert a controlled situation into an emergency.

## Conclusions

Emergency management begins with early recognition of physiologic instability and ductal dependency, followed by immediate stabilization using prostaglandin E1, ventilatory support, and inotropic therapy as indicated. Once hemodynamic stability is achieved, patients are rapidly assigned to a defined intervention window, and palliative catheter-based or surgical therapy is pursued without delay. This time-based strategy reduces clinical indecision and prevents deterioration that may occur during prolonged diagnostic evaluation. There is often a tendency to delay intervention because congenital heart disease is perceived as a long-standing condition. However, severity stratification based on physiologic compromise is essential, and there is a growing need for global guidelines that define disease severity and timing of intervention in congenital heart disease, analogous to the “*door-to-balloon time*” concept in percutaneous coronary intervention.

## Data Availability

The original contributions presented in the study are included in the article/Supplementary Material, further inquiries can be directed to the corresponding author.
